# Transdermal Vaccination with the Matrix-2 Protein Virus-like Particle (M2e VLP) Induces Immunity in Mice against Influenza A Virus

**DOI:** 10.3390/vaccines9111324

**Published:** 2021-11-15

**Authors:** Kimberly Braz Gomes, Sucheta D’Sa, Grace Lovia Allotey-Babington, Sang-Moo Kang, Martin J. D’Souza

**Affiliations:** 1 Center for Drug Delivery Research, Vaccine Nanotechnology Laboratory, Mercer University, Atlanta, GA 30341, USA; sucheta.dsa@gmail.com (S.D.); glallotey-babington@ug.edu.gh (G.L.A.-B.); dsouza_mj@mercer.edu (M.J.D.); 2 Center for Inflammation, Immunity & Infection, Institute for Biomedical Sciences, Georgia State University, Atlanta, GA 30303, USA; skang24@gsu.edu

**Keywords:** virus-like particles (VLPs), transdermal, influenza A virus

## Abstract

In this study, our goal was to utilize the extracellular domain matrix-2 protein virus-like particle (M2e VLP) that has been found to be highly conserved amongst all strains of influenza and could serve as a potential vaccine candidate against influenza. Previous studies have demonstrated that the VLP of the M2e showed increased activation of innate and adaptive immune responses. Therefore, to further explore its level of efficacy and protection, this vaccine was administered transdermally and tested in a pre-clinical mouse model. The M2e VLP was encapsulated into a polymeric matrix with the addition of Alhydrogel^®^ and Monophosphoryl Lipid-A (MPL-A^®^), together referred to as AS04. The M2e VLP formulations induced IgG titers, with increased levels of IgG1 in the M2e VLP MP groups and further elevated levels of IgG2a were found specifically in the M2e VLP MP Adjuvant group. This trend in humoral immunity was also observed from a cell-mediated standpoint, where M2e VLP MP groups showed increased expression in CD4^+^ T cells in the spleen and the lymph node and high levels of CD8^+^ T cells in the lymph node. Taken together, the results illustrate the immunogenic potential of the matrix-2 protein virus-like particle (M2e VLP) vaccine.

## 1. Introduction

The influenza virus is responsible for 3 to 5 million cases of respiratory tract infections accounting for approximately 500,000 deaths each year [1]. Influenza outbreaks that have occurred in the past few decades show that immunity against seasonal influenza does not provide protection against pandemic strains of the virus [2].

The best method for prevention of influenza virus infection is vaccination [3]. There are over 10 licensed vaccines on the market. However, these vaccines suffer from a variety of limitations. One such limitation is the time taken to produce the vaccines [4]. Most of the vaccines incorporate inactivated or attenuated forms of the virus, which are still being produced in embryonic chicken eggs, all of which takes about six months [4]. This poses a major challenge in times when large amounts of the vaccine are required or in the middle of a pandemic where there is no availability of the vaccine to prevent infection [4]. Amongst the approaches adopted to overcome this challenge is the use of recombinant proteins as antigens.

The main proteins of the influenza virus are hemagglutinin (HA), neuraminidase (NA), matrix-1 protein (M1) and matrix-2 protein (M2). The major glycoproteins HA and NA have a mutation rate caused by antigenic drift, resulting in new strains of the virus on an annual basis [3]. Thus, vaccines need to be modified so that they include the circulating strains for the following influenza season [3]. Recent findings have demonstrated that the extracellular domain of the M2 protein (M2e) located within the membrane of the virus is highly conserved across the various strains of influenza and could serve as a basis for a potential universal vaccine candidate [5]. However, its small nature has provided a significant challenge in generating an immune response in vivo [5]. Fortunately, in the last few decades, advances in recombinant technology have led to the development of novel systems such as subunit vaccines that incorporate virus-like particles (VLPs) [6]. The first VLP vaccine was marketed for Hepatitis B in 1986, followed by a VLP vaccine against the human papilloma virus (HPV) in 2006 [7]. VLPs are multiple repeats of a protein or antigen that resemble the native form and organization of the virus (minus its genome), and is therefore a safer candidate for use in a vaccine [7]. The nature of VLPs allow for presentation of antigenic proteins that can enhance antibody production leading to improved immune responses [7]. To improve the immunogenicity of the M2e protein, multiple tandem repeats of the protein self-assemble forming a M2e virus-like particle (VLP).

Studies investigating the M2e VLP as a potential vaccine candidate for influenza have shown promise. However, similar to traditional vaccines, the M2e VLP fails to produce the magnitude of immunity sufficient for protection. The development of synthetic vaccines has revolutionized the delivery of antigens and resolves many of the issues traditional vaccines suffer from [8]. Advances in polymer chemistry has allowed for the advancement of vaccine construction with the ability to encapsulate antigens into particulate form [8]. In the past decade, particulate antigens have an advantage over soluble antigens in that they mimic the nature of the pathogen and are taken up better by antigen presenting cells (APCs), leading to activation of multiple immune pathways and thus a more effective immune response [9]. Particulate antigens have a prolonged release period and can be delivered in higher doses in comparison to soluble antigens [9]. Incorporation of the M2e VLP into a polymeric delivery system was utilized to deliver a well-defined immunological payload and further enhance immunogenicity.

To generate a dynamic immune response, vaccines must often meet two main criteria: they should be antigen-specific and induce long-term protection. To meet these criteria, compounds known as adjuvants are used to enhance activation of the immune system [10]. Research has shown that aluminum-based adjuvants enhance immunogenicity by producing a depository at the site of administration allowing for sustained release of the antigen [11]. This sustained release mechanism allows for increased interface between the antigen and cells of the immune system [11]. In addition to aluminum-based adjuvants, TLR ligands have been shown to improve immunogenicity of vaccines and have been widely accepted and used. One such example is MPL-A^®^ that generates resistance towards viral infections by modulation of cytokine release [7]. MPL-A^®^ was quickly furthered through clinical investigation due to its ability to induce mucosal immune responses and specifically initiate Th-1 responses following viral infections [7,11].

The immune system’s response to a vaccine is also highly dependent on the route of administration [4]. The intramuscular (I.M.) route has been the conventional route for delivery of vaccines due to the minimal adverse effects and high immunogenicity profile of vaccines delivered through this route [4]. Intramuscular injection causes inflammation at the site, which rapidly recruits immune cells, following which the antigen slowly disperses from the site and allows for the development of immunity [4]. In recent years, needle-free vaccines have gained traction as they offer several advantages, some of which include reduction of pain associated with delivery, reduction of blood-borne transmission of diseases through needles, alternative method for delivery of antigens (which cannot be delivered through conventional routes), and self-administration, negating the need for trained professionals [4]. Some new needle-free strategies include jet injectors and microneedles [12].

Microneedles have received increased attention for the delivery of vaccines, particularly through the skin [12]. Microneedles consist of an array of projections which is then placed onto the skin, causing short-term mechanical disruption, allowing the applied drug to reach the epidermis or dermis layers of the skin [13]. The lengths of the needles are short enough to not hit nerve endings, and long enough to penetrate the epidermis or dermis layers [12]. These skin layers are rich in APCs, known as Langerhans cells (LCs) in the epidermis and dermal dendritic cells in the dermis which can activate T and B lymphocytes inducing an immune response and is therefore an excellent route for delivery of vaccines [12].

In this study, we evaluated the immunogenicity of various M2e VLP vaccine formulations administered transdermally in a pre-clinical mouse model for influenza.

## 2. Materials & Methods

### 2.1. Construction of M2e VLP

The 5xM2e VLP (virus-like particle) contains heterologous tandem repeats of two human M2e (SLLTEVETPIRNEWGSRSN), swine M2e (SLLTEVETPTRSEWESRSS), type I avian M2e (SLLTEVETPTRNEWESRSS), and type II avian M2e (SLLTEVETLTRNGWGCRCS), as previously described [12]. The 5xM2e and an oligomer-stabilizing domain GCN4 fusion protein was linked to the transmembrane (TM) cytoplasmic domain of hemagglutinin (HA) to enhance the incorporation of M2e tandem repeats into VLP as previously described [12]. Briefly, to obtain VLPs, Sf9 cells were coinfected with recombinant baculoviruses expressing influenza virus M1 and 5xM2e protein, and cultured in SF900-II serum-free medium at 27 °C. Culture supernatants were collected by centrifugation at 3000 rpm for 20 min to remove cells [12]. The collected supernatants containing influenza VLP were further spun by ultracentrifugation at 30,000 rpm for one hour (h) [12]. The 5xM2e VLP pellets were resuspended in phosphate-buffered saline (PBS) and VLP vaccines were further purified by ultracentrifugation with sucrose gradient (20%, 30%, 60%) at 30,000 rpm for 1 h at 4 °C [12]. The 5xM2e VLP was adsorbed onto formvar/carbon-coated copper grids (Electron Microscopy Sciences, Fort Washington, PA, USA) for 15 min [12]. The transmission electron microscopy picture of the VLPs consisting of M1 and 5xM2e showed spherical particles in a range of 100–180 nm [12].

### 2.2. Preparation of Influenza M2e VLP Particulate Vaccine

The M2e VLP micro particulate vaccine consisted of a polymer matrix with the following components (*w*/*w*): 35% cellulose acetate phthalate dispersion (Aquacoat^®^ CPD, Colorcon, Inc., Indianapolis, IN, USA), 22% hypromellose acetate succinate (HPMCAS), 30% ethyl cellulose dispersion (Aquacoat^®^ ECD, Colorcon, Inc., Indianapolis, IN, USA), 4% trehalose, 4% chitosan and 5% antigen and adjuvant (M2e VLP + MPL-A^®^ + Alhydrogel^®^). First, CPD stock suspension was diluted to 3% by adding 1.5 g of stock to 50 mL of deionized (DI) water under stirring (50 rpm) to dissolve and adjusted to a final pH of 6.0 using 1 N sodium hydroxide (NaOH). Similarly, in a separate 50 mL beaker, 0.3 g of HPMCAS was added to 50 mL of DI water adjusted to a final pH of 8.0 also using 1 N sodium hydroxide (NaOH). To prepare 0.1% *w/v* solid content (0.1 g in 100 mL), in a 100 mL beaker, 50 mL of DI water was first added followed by the addition of 17 mL of CPD, 3.67 mL of HPMCAS and 111 μL of EC and adjusted to a pH of 7.0 under continuous stirring. 4 mg of chitosan was then added. The antigen:adjuvant ratio consisted of VLP:MPL-A^®^:Alhydrogel^®^ at a ratio of 1:2.5:5. Separately, 909 μg of M2e VLP was adsorbed onto 2.94 mg of Alhydrogel^®^ for 1 h, followed by the addition of 47 mg of MPL-A^®^ (5 mg total *w*/*w*). This antigen:adjuvants mixture was then added to the polymer mixture followed by 4 mg of trehalose. Finally, 0.01% *v*/*v* of Tween 20 was added to formulation. The total volume of the mixture was q.s. to 100 mL and the formulation was spray dried into particulates using the Buchi B290 spray dryer.

### 2.3. Immunization of Mice

**Figure 1:**
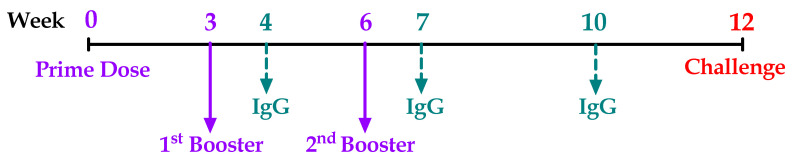
Immunization schedule. Mice were immunized with a prime-boost regimen at weeks 0, 3, and 6. Antibody levels were measured in serum collected from mice at weeks 4, 7, and 10. Mice were challenged with live influenza virus strain A/Philippines/2/82 (H3N2) (4 × 10^3^ PFU) at week 12.

For animal experiments, four- to six-week-old male C57BL/6 mice (Charles River Laboratories, Wilmington, MA, USA) were used. The details of the study are listed in Table 1 and Figure 1. One prime (Week 0) and two booster (Week 3, 6) doses were administered to mice intramuscularly (I.M.) or transdermally (T.D.) using the AdminPatch^®^ 1200 microneedle array. The AdminPatch^®^ 1200 microneedle array was first used to create pores on the skin of the C57BL/6 mice. Transdermal vaccination was done using a syringe where the microparticle formulation was first suspended, then loaded and applied onto the treated skin. For intramuscular administration, 0.5 μg of a monovalent inactivated H1N1 (A/California/07/2009) Influenza A vaccine was administered. For transdermal administration, 5 μg of M2e VLP was added to 200 μL of phosphate buffered saline (PBS) upon administration for both M2e VLP suspension and particulate (MP) groups. The adjuvant group received 5 μg of M2e VLP, 12.5 μg MPL-A^®^ and 25 μg of Alhydrogel^®^. Mice were then evaluated for antibody responses at weeks 1, 4, 7 and 10, challenged at week 12 and euthanized at week 14, following which lung, spleen, lymph nodes and bone marrow were collected for evaluation of T cell responses and viral titer.

### 2.4. Determination of Antibody Responses

Blood samples were collected before immunization and every three weeks (Weeks 1, 4, 7 and 10). For collection of serum, blood was left to clot for 20 min at room temperature, then centrifuged at 1500 G for 10 min at 4 °C. Samples were stored at −20 °C until analysis. Specific serum antibody for M2e was assessed using ELISA. Coating antigen included the M2e peptide or the inactivated virus (for H1N1 specific antibodies-control) that were added to microtiter plates at a concentration of 0.2 μg/well. The plates were incubated at 4 °C overnight. The plates were washed three times with PBST (PBS + 0.1% Tween 20) and then a blocking buffer (PBS + 3% BSA) was added to the plates and incubated for 2.5 h at room temperature on a rotator at 60 rpm. Following the blocking step, the plates were washed three times with PBST (PBS + 0.1% Tween 20^®^). Serum samples were left to thaw on ice and ten-fold serially diluted in PBS starting at 1:10 for a total of 10 dilutions. The diluted samples were then added to the plates at a volume of 50 μL/well and incubated at 37 °C for 1.5 h. The plates were then washed three times with PBST (PBS + 0.1% Tween 20^®^). Horseradish peroxidase (HRP) conjugated goat anti-mouse IgG, IgG1 and IgG2a were used as secondary antibodies at a dilution of 1:2000, added at a volume of 50 μL/well and incubated at 37 °C for 1.5 h. The plates were then washed three times with PBST (PBS + 0.1% Tween 20^®^) to determine the total amount of antibody and antibody isotypes. The substrate 3, 3′, 5, 5′–Tetramethylbenzidine (TMB) was used at a volume of 50 μL/well, incubated at room temperature for 3 min, followed by the addition of 50 μL/well of stop solution (0.3 M sulfuric acid (H_2_SO_4_)) for detection of color. The optical density was taken at 450 nm on the Biotek Synergy plate reader. The cut-off value (COV) of the OD was established as the average + 2 standard deviations of serum from control mice. For a given sample in each group, the highest dilution of sera that had an OD above the COV was considered as a positive titer. For each group, the antibody titers were averaged to give the geometric mean titer, and compared between weeks.

### 2.5. Challenge with A/Philippines/2/82 (H3N2) Influenza Virus

Mice were intranasally challenged with A/Philippines/2/82 (H3N2) (4 × 10^3^ PFU) live influenza virus on week 12. The complete immunization schedule is shown in Figure 1. All animal experiments were approved by Mercer University IACUC review board and conducted under the guidelines of Mercer University IACUC. (Animal protocol #A1504008).

### 2.6. Evaluation of T Cell Responses

Animals were sacrificed at week 14 and the primary (bone marrow) and secondary lymphoid organs (spleen and lymph node) were collected from all five groups (*n* = 6 mice/group) in incomplete medium (RPMI 1640). Bone marrow was collected from the femur and tibia and placed in incomplete RPMI medium. For removal of red blood cells (RBCs), a water lysis was carried out using 900 μL of sterile filtered water and 100 μL of 10× PBS and centrifuged at 1500 rpm for 5 min. The cells were then plated in petri dishes at 1 × 10^6^ cells per plate in 10 mL of complete RPMI 1640 medium (R10) + IL-2 overnight. Single cell suspensions of the secondary lymphoid organs were made using a 40 μm cell strainer (purchased from Fischer Scientific, Waltham, MA, USA). Lymph node cell suspensions were centrifuged for 10 min at 3500 rpm and then added to a T-25 flask in complete R10 medium + IL-2 overnight. For removal of red blood cells from the spleen, ACK lysis buffer was added to the splenocytes and centrifuged for 10 min at 3500 rpm five times. Splenocytes were then cultured in R10 + IL-2 overnight. Single cell suspensions of the lymph node and spleen were stained with fluorescently labeled antibodies for detecting T cell phenotypes, CD4^+^ (FITC anti-mouse, BD Biosciences, Franklin Lakes, NJ, USA) and CD8a+ (PE anti-mouse, BD Biosciences, Franklin Lakes, NJ, USA), quantified using flow cytometry (BD AccuriTM C6 flow cytometer).

### 2.7. Lung Viral Titers

The whole lung tissue was isolated after challenge and homogenates were prepared and stored at −80 °C for analysis. Madin-Darby Canine Kidney (MDCK) epithelial cells were used as a propagation system for growth of the influenza virus. The MDCK cells were seeded at 1 × 10^6^ on a six-well plate in Eagles Minimum Essential Medium (EMEM) supplemented with 10% FBS and left for 24 to 48 h until 90% confluency. The lung viral titers were determined by adding serial dilutions of the supernatant from the lung homogenates to the MDCK cells for 1 h at 37 °C, shaking the plates gently every 15 min. The cells were then washed twice and a 3% agarose overlay was added to the cells and left for 15 min at room temperature to solidify. The cells were then placed in the incubator at 37 °C for two days for plaque formation. At day two post-infection, cells were fixed with 3% formaldehyde at room temperature for 1 h and then washed gently with water. The cells were then stained for 5 min with 0.5% crystal violet and plaques were visualized. 

### 2.8. Analytical Tests

All statistical analyses were conducted using the Graphpad Prism 7 software. For group comparisons, a one-way ANOVA was applied. The following *p* values were used, *p* > 0.05 (non-significant), *p* < 0.05 (*), *p* < 0.01 (**) and *p* < 0.005 (***). Error bars are indicative of standard deviation of uncertainty.

## 3. Results

### 3.1. Characterization and Immunogenicity of M2e VLP Micro Particulate Vaccine

#### 3.1.1. Microparticle Size and Yield

The microparticle yield was found to be 92% and the encapsulation yield was around 84% with a size of approximately 1.85 μm for the M2e VLP MP. The microparticle yield was found to be 90% and the encapsulation yield was around 88% with a size of approximately 2.49 μm for the M2e VLP MP + MPL-A^®^ + Alhydrogel^®^. The yield following spray drying is important when considering the number of losses during processing. The encapsulation yield is important when studying the amount of VLP that is encapsulated and the size of the M2e VLP MP is critical for uptake in antigen presenting cells that better recognize sizes that range between 1 to 3 μm [14].

#### 3.1.2. Serum IgG Antibodies against the M2e VLP

**Figure 2:**
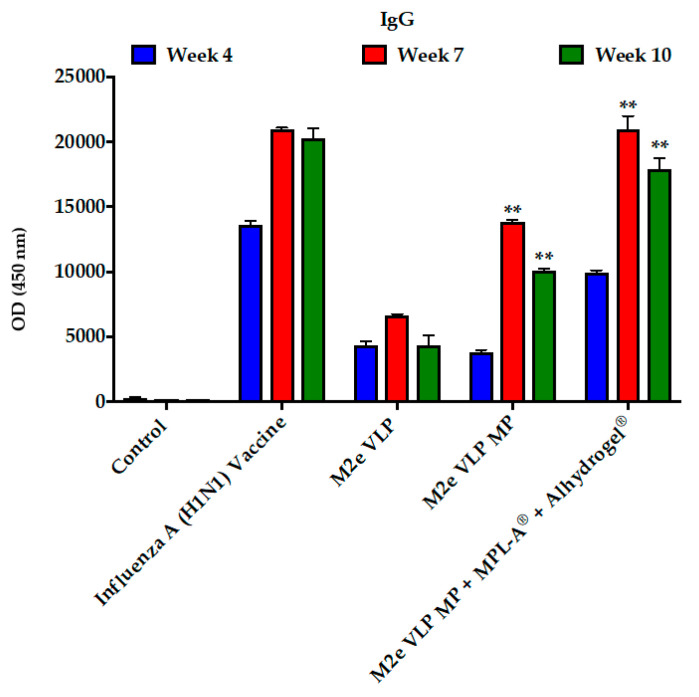
Antibody response. Serum IgG recognizing M2e VLP in immunized mice. Mice (*n* = 6) receiving Influenza A (H1N1) vaccine were immunized intramuscularly with 0.5 μg of H1N1 antigen and mice receiving M2e VLP were immunized transdermally with 5.0 μg of VLP using the AdminPatch^®^ 1200 microneedle array. The adjuvant group received 5 μg of M2e VLP, 12.5 μg MPL-A^®^ and 25 μg of Alhydrogel^®^. **, *p* < 0.01.

**Figure 3:**
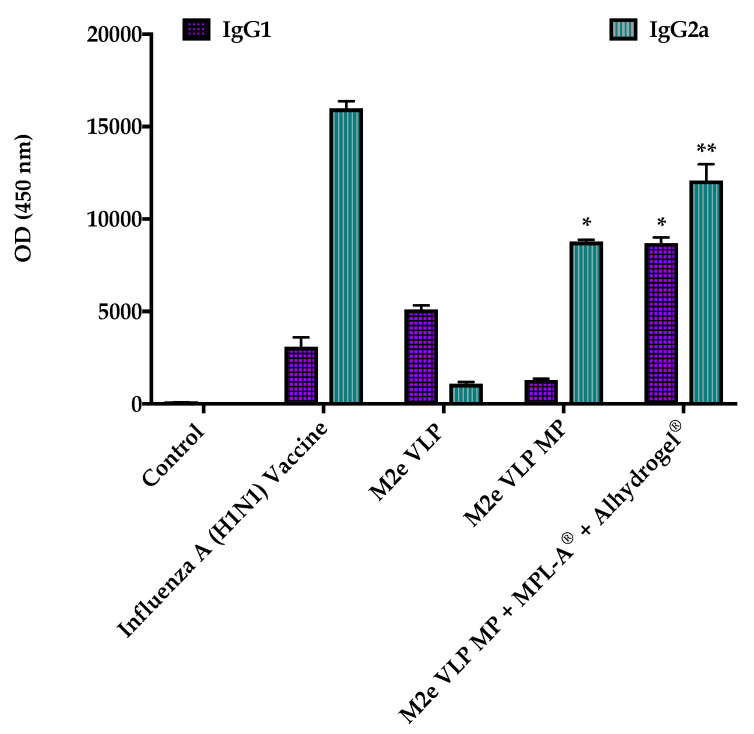
Isotypes of influenza specific antibodies in serum of immunized mice. Serum IgG1 and IgG2a recognizing M2e VLP. *, *p* < 0.05, **, *p* < 0.01.

The efficacy of the M2e VLP vaccine was assessed by administration of the following: (1) Control (PBS), (2) 0.5 μg of monovalent inactivated influenza virus vaccine (H1N1), (3) 5 μg of M2e VLP in suspension, (4) 5 μg of M2e VLP in microparticulates (MP) and (5) 5 μg of M2e VLP in microparticulates (MP) + MPL-A^®^ + Alhydrogel^®^. A prime and two booster doses were administered and blood samples were collected at weeks 1, 4, 7, and 10 for assessment of antibody responses to the M2e VLP using ELISA (Figure 2). The inactivated influenza virus vaccine (H1N1) elicited high titers at week four. All formulations illustrated antigen-specific antibody responses (IgG) (Figure 2). M2e VLP, M2e VLP MP and M2e VLP MP + MPL-A^®^ + Alhydrogel^®^ showed elevated levels of IgG beginning at week 7, demonstrating that the M2e VLP is immunogenic (Figure 2). The adjuvant group, M2e VLP MP + MPL-A^®^ + Alhydrogel^®^, showed higher IgG responses compared to the M2e VLP MP and M2e VLP formulations (Figure 2). The control mice illustrated very low titers compared to all other groups. Total IgG levels and subclass titers showed strong correlation in the adjuvant vaccine group. Isotypes of IgG, IgG1 and IgG2a were evaluated. IgG1 antibodies were elevated in the M2e VLP MP + MPL-A^®^ + Alhydrogel^®^ (Figure 3). There were also levels of IgG 1 present in the M2e VLP and inactivated H1N1 vaccinated groups. The adjuvant group showed increased levels of Th1 related subclass IgG2a compared to M2e VLP MP and M2e VLP formulations. 

#### 3.1.3. M2eVLP Induced Protection Following Challenge

**Figure 4:**
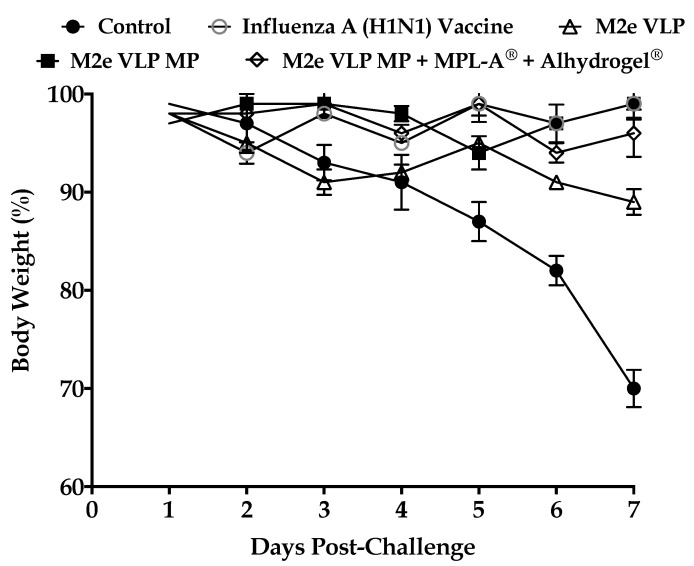
Body weight changes observed in mice eight days’ post challenge. The control group showed substantial decrease in body weight percentage.

All mice were challenged with live influenza virus strain A/Philippines/2/82 (H3N2) (4 × 10^3^ PFU). Decrease in body weight was used as an indication of viral infection. Following challenge, all control mice showed more than 25% loss in body weight (Figure 4). There was also a significant decrease in body weight (15%) in the M2e VLP group. All other groups showed slight fluctuations in body weight. However, no significant loss in body weight was observed. 

#### 3.1.4. M2e VLP Specific CD4^+^ T Cells

**Figure 5:**
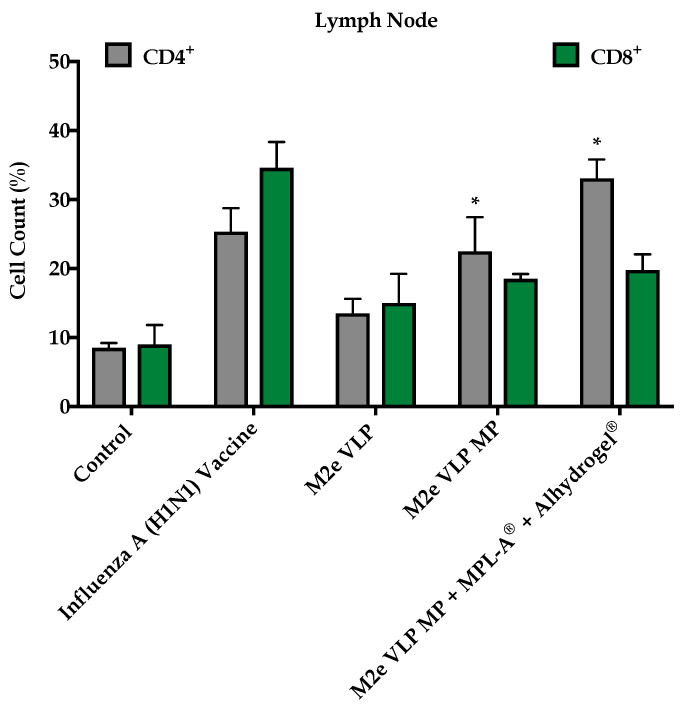
T cell phenotype analysis. CD4^+^ and CD8^+^ T cell populations in the lymph node (LN) using fluorescently tagged antibodies, measured by flow cytometry *, *p* < 0.05.

**Figure 6:**
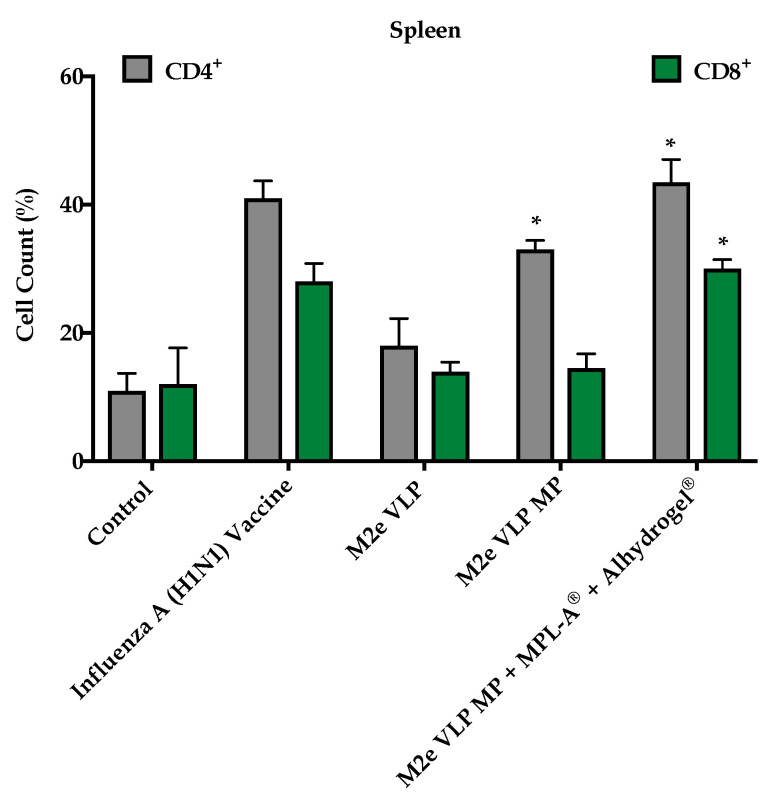
T cell phenotype analysis. CD4^+^ and CD8^+^ T cell populations in the spleen using fluorescently tagged antibodies, measured by flow cytometry. *, *p* < 0.05.

**Figure 7:**
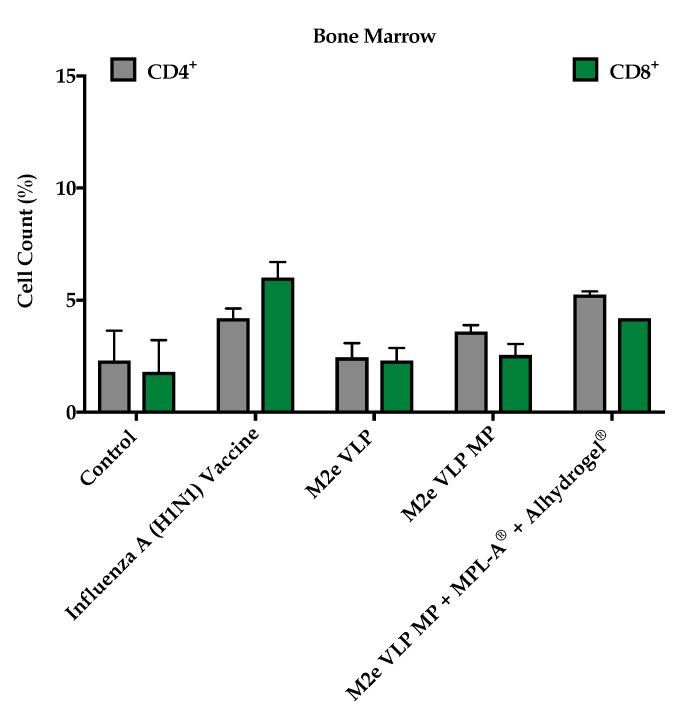
T cell phenotype analysis. CD4^+^ and CD8^+^ T cell populations in the bone marrow using fluorescently tagged antibodies, measured by flow cytometry.

To investigate effector T cells following challenge, CD4^+^ and CD8^+^ T cell populations were assessed in bone marrow and secondary lymphoid organs such as the spleen and lymph nodes. Mice that were immunized with the M2e VLP MP and M2e VLP MP + MPL-A^®^ + Alhydrogel^®^ had high expression of CD4^+^ T cells in the spleen and the lymph node (Figure 5 and Figure 6). The M2e VLP MP + MPL-A^®^ + Alhydrogel^®^ showed high levels of CD8^+^ cells in the lymph node (Figure 5 and Figure 6). There were very low levels of effector CD4^+^ and CD8^+^ T cells present in the bone marrow (Figure 7).

#### 3.1.5. Viral Titer in Lung

**Figure 8:**
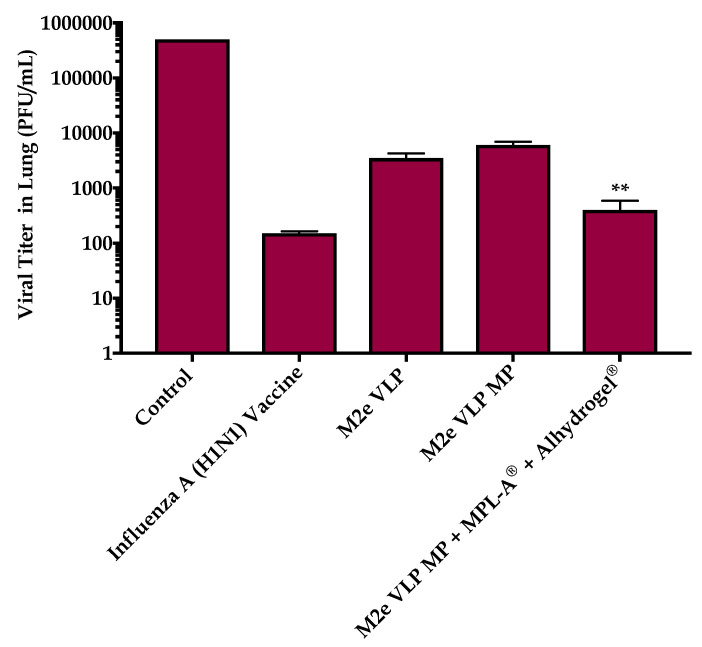
Plaque assay conducted in Madin-Darby Canine Kidney (MDCK) epithelial cells plated at a density of 1 × 10^6^ cells. Lung homogenates collected from all immunized groups were used as viral stock samples and added to the cells. The viral titers are expressed as plaque forming units/mL. **, *p* < 0.01.

The mice were sacrificed eight days following challenge and lung homogenates were prepared. Madin- Darby Canine Kidney (MDCK) epithelial cells were infected with lung samples and the viral titer was quantified by the number of plaque forming units in a given volume (PFU/mL). Figure 8 shows the viral titer in all the vaccinated groups. The viral titer was shown to be 10-fold lower in the M2e VLP MP + MPL-A^®^ + Alhydrogel^®^ vaccinated mice compared to M2e VLP and M2e VLP MP (Figure 8). As expected, the viral load in the control was considerably high.

## 4. Discussion

This study investigated the use of a potential universal M2e VLP particulate as influenza A vaccine and compared its efficacy to the inactivated monovalent H1N1 vaccine. The extracellular domain of the matrix-2 protein (M2e) is a great target due to its conservation among seasonal influenza A strains. The poor immunogenicity of the M2e was improved by using multiple tandem repeats of the protein forming an immunogenic M2e VLP [6]. To further enhance the immune response, the M2e VLP was incorporated into a highly effective delivery system forming an M2e VLP microparticulate (MP) vaccine with the addition of immunostimulatory compounds such as MPL-A^®^ and Alhydrogel^®^.

Data from this study demonstrated that the M2e VLP when encapsulated into a micro particulate matrix (M2e VLP MP) showed increased antibody titers (Total IgG, IgG1, IgG2a) which was further enhanced with the addition of adjuvants (M2e VLP MP + MPL- A^®^ + Alhydrogel^®^). Investigation of effector T cell subsets in lymphatic organs illustrated that groups receiving either of the MP formulations had increased expression in CD4^+^ and CD8^+^ T cells that play an important role in combatting virus during infection. This was further explored in the lung which is the main site of influenza infection. Viral titers studied in the lung confirmed that immunization with the M2e VLP MP + MPL-A^®^ and Alhydrogel^®^ showed decreased lung viral titers and less weight loss following challenge, compared to M2e VLP and M2e VLP MP. However, there was no significance observed between the M2e VLP MP + MPL-A^®^ and Alhydrogel^®^ immunized mice and the H1N1 inactivated influenza A vaccinated group with respect to body weight and viral load (Figure 4 and Figure 8). 

The particulate M2e VLP adjuvanted vaccine is unique in that it allowed for adsorption of the M2e VLP onto Alhydrogel^®^, which was then encapsulated with MPL-A^®^. The size and shape of the particle is a major contributing factor to its capability of stimulating immune responses [15]. Initiating immune responses is dependent on the transport of the antigen to the secondary lymphoid organs [15]. Lymphatic vessels can carry micron-sized particles, however 500 nm–2 μm in size must be transported by APCs and are not able to enter lymph capillaries on their own [16]. Researchers have also demonstrated that microparticles between 1–5 μm are carried to the spleen by APCs where once released, the antigen induces an antibody response [17]. The M2e VLP microparticle vaccine measured around 1–2 μm and based on the data shown, this particle size range is optimum for uptake by APCs through the process of phagocytosis [9]. Once internalized, particles are processed in the endosome of the APCs and presented on MHC Class II for activation of CD4^+^ T cells [18]. Some particles have been found to escape into the cytoplasm, where the antigen is slowly released and presented on MHC Class I, stimulating CD8^+^ T cells [18].

Testing the M2e VLP MP + MPL-A^®^ + Alhydrogel^®^ in C57BL/6 mice illustrated that this vaccine formulation induced antibody responses (Figure 2), similar to that of the monovalent inactivated (H1N1) influenza virus vaccine. In addition, it was also observed that the M2e VLP MP with adjuvants demonstrated a significantly higher antibody titer compared to M2e VLP MP alone (Figure 2). Adjuvants, specifically Alhydrogel^®^, are known to enhance the immunogenicity of a vaccine by forming a depot at the site of administration which induces cytokine release and activates APCs [11]. The process by which the adjuvanted M2e VLP MPs were formulated could have corresponded to the increased antibody titers observed in this study. Research has shown that adsorption of an antigen onto aluminum compounds stimulates more robust immune responses [11]. The M2eVLP MP adjuvant group also included MPL-A^®^, in addition to Alhydrogel^®^. MPL-A^®^ is a well-known TLR-4 ligand that can program a specific immune response by activating the NFκB pathway that leads to the expression of cytokines [11]. The difference in responses produced by Alhydrogel^®^ and MPL-A^®^ serves as a basis, that together the two adjuvants can work synergistically with each other and have been marketed in combination as AS04 [11]. Previous studies have demonstrated that AS04, compared to aluminum salts alone induces immune responses that are long-lasting [11].

This study illustrated that the M2e VLP vaccine with the addition of AS04 (MPL-A^®^ and Alhydrogel^®^) can be protective and may play a role in protecting against H3N2 (A/Philippines/2/82) virus used in this study. The higher levels of IgG1 in the M2e VLP group versus IgG2a in the M2e VLP MP and M2e VLP MP + MPL-A^®^ and Alhydrogel^®^ groups demonstrate that the M2e specific IgG2a is more protective than IgG1 (Figure 3). Furthermore, this increase in IgG2a seen in the M2e VLP MP group with adjuvants surpasses that of the mice immunized with M2e VLP MP and may also contribute to better elimination of virus-infected cells compared to M2e VLP alone. IgG2a is known to be extremely important in the defense against influenza, as a study showed that SCID mice immunized with IgG2a monoclonal antibodies against M2e were protected following challenge with live influenza virus [19]. The M2e VLP MP vaccinated groups produced better antibody responses than the VLP group alone. Furthermore, the adjuvanted M2e VLP MP group showed superior antibody responses compared to VLP alone (Figure 3). Taken together, our study illustrates that the adjuvanted M2e VLP MPs elicits a stronger overall humoral response.

In addition to M2e specific antibodies, protection against influenza is highly dependent on CD4^+^ T cells and recovery following influenza infection is highly dependent on CD8^+^ T cells [20]. In this study, enhanced CD4^+^ and CD8^+^ T cell responses were observed in groups that were immunized with M2e VLP MP and M2e VLP + adjuvants. In particular, the lymph node and spleen showed significantly higher levels of CD4^+^ and CD8^+^ T cell subsets, respectively, in the M2e VLP MP and adjuvanted M2e VLP immunized mice, compared to VLP alone (Figure 5 and Figure 6). Protective responses against various influenza subtypes are rooted in the T cells, as the peptides that are known to stimulate T cells tend to be more conserved compared to binding sites for these peptides on antibodies [20].

The lack of heterosubtypic immunity with current influenza vaccines is due to the fact that the CD8^+^ and CD4^+^ T cells tend to be specific for HA and NA from homosubtypic strains [20]. Compared to HA and NA, M2e is highly conserved and the M2e VLP was constructed using heterologous tandem repeats of M2e; therefore, vaccination with the M2e VLP could have potential for heterosubtypic immunity and must be further explored. In this study, only one strain of H3N2 influenza A (A/Philippines/2/82) was used for challenge. Impending studies should consider multiple strains for challenge to thoroughly investigate heterosubtypic immunity. Additionally, only IgG, IgG1 and IgG2a subtypes of antibodies were tested. In future, some of the other subtypes of IgG (IgG2c in the case of C57BL/6 mice) as well as IgA should be measured for confirmation of mucosal immunity that may be relevant to transdermal vaccine delivery. Another shortcoming in this study surrounds the use of restricted T cell markers. Inclusion of various T cell subsets, including T memory should be examined. 

## 5. Conclusions

The current licensed vaccines against influenza are facing numerous challenges associated with production time, antigenic changes, route of administration, etc. Developing an extracellular domain matrix-2 protein virus-like particle (M2e VLP) microparticulate vaccine which is easy to formulate and is stable, immunogenic, safe, and protective could have a huge impact and help circumvent many of these challenges. Our preclinical study showed that an adjuvanted M2e VLP microparticulate vaccine can induce humoral and cellular immunity. The data shown here serves as a foundation for further exploration of a prospective universal vaccine that implicates a potential vaccine strategy against influenza.

## Figures and Tables

**Table 1:** M2e VLP subunit vaccine groups. Mice (N = 6) were immunized with M2e VLP. Control mice received PBS and served as the negative control for all groups, while inactivated influenza virus (H1N1) served as the positive control for all groups.

Group (N = 6)	Dose (μg)	Route (I.M.—Intramuscular), (T.D.—Transdermal)
**Control (PBS)**	-	-
**Inactivated Influenza virus (H1N1)**	0.5	I.M
**M2e VLP**	5	T.D.
**M2e VLP (MP)**	5	T.D.
**M2e VLP MP + MPL-A^®^ + Alhydrogel^®^**	5	T.D.

## Data Availability

Data will be made available upon reasonable request.
